# USING REAL-WORLD EVIDENCE TO SUPPORT THE FDA REGULATORY APPROVAL: METHODOLOGICAL CONSIDERATIONS

**DOI:** 10.1093/geroni/igac059.770

**Published:** 2022-12-20

**Authors:** Kevin Lu, Patricia Slattum

## Abstract

Evidence from clinical trials has traditionally been used to support regulatory drug approval, whereas real-world evidence (RWE) has been used for post-marketing surveillance studies. With the enaction of the 21st Century Cures Act, the Food and Drug Administration (FDA) is evaluating the potential use of RWE to support new indications for an approved product or to satisfy post-approval study requirements. Yet the substantial evidence standard remains unchanged for FDA approval. To date, the FDA has issued guidelines or frameworks for using RWE for regulatory decisions. However, many methodological challenges remain unanswered when using RWE in gerontology research. This Pharmaceutical Care and Outcomes Research Interest Group-sponsored symposium consists of leading experts in the country to address these problems. The four papers in this symposium will address methodological challenges that are unique in gerontology research. Specifically, The first paper will introduce the FDA drug evaluation process, focusing on the key considerations for evaluating RWE in regulatory decision making; the second paper will discuss possible challenges related to data sources from which RWE are generated in aging research; the third paper will provide a case example of some of the challenges in using RWD sources to generate RWE in the Alzheimer’s Disease setting; and the fourth paper will provide recommendations for improving gerontology research based on RWE going forward.

